# The Effect of Axial Length Elongation on Corneal Biomechanical Property

**DOI:** 10.3389/fbioe.2021.777239

**Published:** 2021-12-02

**Authors:** Guihua Liu, Hua Rong, Ping Zhang, Yu Xue, Bei Du, Biying Wang, Jiamei Hu, Zhi Chen, Ruihua Wei

**Affiliations:** ^1^ Tianjin Key Laboratory of Retinal Functions and Diseases, Tianjin Branch of National Clinical Research Center for Ocular Disease, Eye Institute and School of Optometry, Tianjin Medical University Eye Hospital, Tianjin, China; ^2^ NHC Key Laboratory of Myopia, Eye Institute and Department of Ophthalmology, Eye and ENT Hospital, Fudan University, Shanghai, China; ^3^ Key Laboratory of Myopia, Shanghai Research Center of Ophthalmology and Optometry, Chinese Academy of Medical Sciences, Shanghai, China

**Keywords:** CorVis ST, corneal biomechanics, anterior segment, axial length, myopia

## Abstract

**Background:** To investigate the correlation between the corneal biomechanical parameter stress-strain index (SSI) and axial length (AL) in moderately elongated eye (MEE) and severely elongated eye (SEE).

**Methods:** This study included 117 eyes from 117 participants. Among them, 59 (50.4%) had MEE (AL<26 mm) and 58 (49.6%) had SEE (AL≥26 mm). AL was measured using Lenstar LS-900, and central corneal thickness (CCT) and anterior chamber volume (ACV) were measured using Pentacam. SSI was measured *via* corneal visualisation Scheimpflug technology (Corvis ST). Kolmogorov-Smirnov test, Student’s *t*-test, and Pearson and partial correlation analyses were used for statistical analyses.

**Results:** The mean (±SD) SSI was 1.08 ± 0.15 in the MEE group and 0.92 ± 0.13 in the SEE group (*p* < 0.01). SSI was positively correlated with age (MEE: *r* = 0.326, *p* < 0.05; SEE: *r* = 0.298, *p* < 0.05) in both groups; it was negatively correlated with AL (*r* = −0.476, *p* < 0.001) in the MEE group but not in the SEE group (*p* > 0.05). CCT was negatively correlated with AL (*r* = −0.289, *p* < 0.05) and ACV positively correlated with AL (*r* = 0.444, *p* < 0.001) in the MEE group. Neither CCT nor ACV was correlated with AL (*p* > 0.05) in the SEE group.

**Conclusion:** Corneal biomechanical parameter SSI, which represents the stiffness of corneal tissue, was lower in the SEE group than in the MEE group. When analyzed separately, SSI was negatively correlated with AL in the MEE group, but not in the SEE group, which may provide insight into different ocular growth patterns between lower myopia and higher myopia.

## Background

Myopia is a common ocular disorder worldwide, and its incidence is increasing with an alarming rate. A total of 49.8% of the world population, i.e., approximately 4,758 million, will be affected by myopia by the year 2050 (Holden et al., 2016). High myopia, defined by a myopic refractive error worse than -5.00D or -6.00D (Flitcroft et al., 2019), is accompanied by a severely elongated eye along with other ocular component changes, which potentially leads to a higher chance of complications, such as retinal detachment, glaucoma (Haarman et al., 2020), scleral thinning, and localized ectasia of the posterior sclera (Rada et al., 2006).

Axial elongation is an important indicator of the development of myopia and a major factor leading to visual impairment. Previous studies have shown that eyes with axial length (AL) of 26 mm or greater had significantly higher risk for visual impairment than eyes with AL shorter than 26 mm in Europeans under 60 years of age (Tideman et al., 2016). In severely elongated eyes, scleral thickness and rigidity are significantly reduced (Sergienko and Shargorogska, 2012; Jonas et al., 2019). Some researchers believe that the eyeball is an integral organ, and the anterior segment parameters, including corneal biomechanics, are related to those of the posterior sclera (Chansangpetch et al., 2017; Bataille et al., 2021). Previous studies found that high myopia can cause corneal biomechanical changes (Moshirfar et al., 2019). Some studies found a negative correlation between corneal biomechanics and AL (Long et al., 2019; Tubtimthong et al., 2020; Yu et al., 2020) and suggested that measuring corneal biomechanics can predict axial elongation (Haseltine et al., 2012; Zadnik et al., 2015; Long et al., 2019; Wu et al., 2019). In contrast, other studies suggested that corneal biomechanics is independent of the severity of refractive error (Lim et al., 2008; Inceoglu et al., 2018). Therefore, exploring the relationship between corneal biomechanical parameters and axial elongation, especially in different refractive groups, can help develop suitable clinical interventions for myopia.

Regarding corneal biomechanical measurement technology, the new parameter stress-strain index (SSI) provided by corneal visualization Scheimpflug technology (Corvis ST) can reflect corneal stiffness without being affected by central corneal thickness (CCT) or intraocular pressure (IOP) (Eliasy et al., 2019). One of our previous studies found that the SSI was negatively correlated with AL, but high myopia was not included for that study (Liu et al., 2020). However, the extent of myopia may influence corneal biomechanics (Yu et al., 2020). For example, at the outset, corneal stiffness are weakened as the eye elongates in physiological processes (Khokhar et al., 2018; Han et al., 2020; Liu et al., 2020). When the elongation enters the pathological stage, corneal stiffness may tend to stabilize (Larsen, 1971; Khokhar et al., 2018).

In the current study, we first expanded the AL range of the study participants and divided them into the moderately elongated eye (MEE) and severely elongated eye (SEE) groups according to their AL (<26 mm and ≥26 mm, respectively). Corneal stiffness parameter SSI and its influencing factors were then compared between the two groups. The purpose of this study was to investigate the relationship between AL and corneal stiffness and to provide an explanation for the controversies raised in previous studies.

## Methods

### Subjects

This prospective study included 117 participants who were consecutively recruited from May to October 2020 at Tianjin Medical University Eye Hospital. Only participants without ocular diseases such as corneal pathology, refractive surgery history, keratoconus, allergic eye disease, uveitis, glaucoma, history of intraocular surgery, or any significant systemic illness were included. Written informed consent was obtained from all enrolled participants or their parents or guardians if the participants were under 16 years old. All study procedures adhered to the tenets of the Declaration of Helsinki and were approved by the ethics committee of Tianjin Medical University Eye Hospital.

The subjects were divided into two groups based on their ALs, with AL greater than or equal to 26 mm defined as SEE, and AL less than 26 mm defined as MEE. All subjects underwent a complete ophthalmic examination, including slit-lamp ophthalmic examination, visual acuity measurement, subjective refraction, fundus examination, and IOP measurement.

### AL Measurement

AL was measured using a non-contact biometer (Lenstar LS-900; Haag-Streit AG, Berne, Switzerland). During the examination, the subjects were instructed to keep both eyes open and fixate on the target. The subjects were allowed to blink their eyes between the measurements to ensure an intact tear film and to avoid potential measurement errors. The results for data analysis were obtained by averaging three repeated measurements in which intrasession differences were no greater than 0.02 mm.

### CCT and Anterior Chamber Volume (ACV)

Pentacam (Oculus, Wetzlar, Germany) was used to measure CCT, corneal curvature, and ACV. All measurements were performed in a dark room. Before examination, the participants blinked their eyes briskly several times and were then asked to fixate on the target and keep their eyes wide open for the scan. Only measurements with an “OK” quality index were saved.

### Corneal Biomechanical Parameters

Corneal biomechanical parameters were measured using Corvis ST (Oculus, Wetzlar, Germany). It is a dynamic and non-contact tonometer equipped with an optical pachymetry function that allows imaging in response to an air puff. The cornea was recorded at 4,330 images per second by using a built-in high-speed camera. The SSI was also recorded using this device. The SSI is a new parameter, which was established to eliminate the interference of IOP and corneal geometry and to estimate the stiffness of a material that differs from the stiffness parameter (SP). The SSI algorithm was based on the prediction of corneal behavior by using finite element numerical modeling simulation of the influence of IOP and Corvis ST air puff on corneal behavior. All measurements were performed by certified technicians. Only measurements with an “OK” quality index were included in the analysis.

### Statistical Analysis

Data were summarized into Microsoft Excel sheets, and statistical analyses were performed using SPSS statistical package 25 (SPSS, IBM, Chicago, IL, United States). The differences in ocular parameters were determined using a paired *t*-test. The Kolmogorov-Smirnov test was used to assess the normal distribution of data. Student’s *t*-test was used to compare the corneal parameters between the two groups. Pearson correlation analysis was used to test the correlation between the SSI and age, while the partial correlation coefficient adjusted for age was used to analyze the correlation between the SSI, CCT, ACV, and AL. A *p* value less than 0.05 was considered statistically significant.

## Results

In total, 117 left eyes of 117 patients who met the study inclusion criteria were analyzed. Among them, 59 (50.4%) were included in the MEE group and 58 (49.6%) in the SEE group. The SSI and age showed a normal distribution (*p* > 0.05) in both groups. The normal distribution of each parameter was summarized in Table 1. The comparison of the parameters of the left and right eyes was summarized in Table 2. Because the SSI showed no significant difference between the contralateral eyes (*p* > 0.05), only the left eyes were included in the subsequent analysis.

**TABLE 1:** Kolmogorov-Smirnov test of parameters.

	MEE		SEE		All patients	
	K-S Z	*p*	K-S Z	*p*	K-S Z	*p*
SSI	0.83	>0.2^a^	0.115	0.056^a^	0.057	>0.2^a^
Age, years	0.79	>0.2^a^	0.107	0.097^a^	0.067	>0.2^a^
AL, mm	0.83	>0.2^a^	0.116	0.051^a^	0.067	>0.2^a^
CCT, μm	0.62	>0.2^a^	0.084	>0.2^a^	0.550	>0.2^a^
ACV, mm^3^	0.72	>0.2^a^	0.095	>0.2^a^	0.070	>0.2^a^

a Normal distribution. SSI, Stress-Strain index; AL, Axial length; CCT, Central corneal thickness; ACV, Anterior chamber volume; K-S Z, Kolmogorov-Smirnov Z value; MEE, moderately elongated eye; SEE, severely elongated eye.

**TABLE 2:** Paired *t*-test comparison of left eye and right eye.

	OD		OS		*p* value
	Mean	SD	Mean	SD	
SSI	0.99	0.19	1.00	0.16	0.405
AL, mm	26.06	2.34	26.09	2.35	0.225
CCT, μm	537.22	32.60	536.92	31.46	0.586
ACV, mm^3^	197.32	34.16	197.66	34.32	0.642

SSI, Stress-Strain index; AL, Axial length; CCT, Central corneal thickness; ACV, Anterior chamber volume; SD, Standard deviation.

The mean age of the subjects was 24.7 ± 8.9 years old and not different between the MEE and SEE groups (*p* = 0.802). The overall AL was 26.09 ± 2.35 mm, being significantly longer in the SEE group (28.07 ± 1.42 mm) than in the MEE group (24.14 ± 1.15 mm) (*p* < 0.001). The overall SSI was 1.00 ± 0.16, being significantly lower in the SEE group (0.92 ± 0.13) than in the MEE group (1.08 ± 0.15) (*p* < 0.001). Data were presented in Table 3.

**TABLE 3:** Comparison of parameters between two groups.

	MEE		SEE		*p* value
	Mean	SD	Mean	SD	
SSI	1.08	0.15	0.92	0.13	<0.001^a^
Age, years	24.88	9.77	24.47	8.06	0.802
AL, mm	24.14	1.15	28.07	1.42	<0.001^a^
CCT, μm	539.49	28.76	534.31	34.03	0.375
ACV, mm^3^	182.97	34.91	212.60	26.59	<0.001^a^

a Statistically significant. SSI, Stress-Strain index; AL, Axial length; CCT, Central corneal thickness; ACV, Anterior chamber volume; SD, Standard deviation; MEE, moderately elongated eye; SEE, severely elongated eye.

**FIGURE 1:**
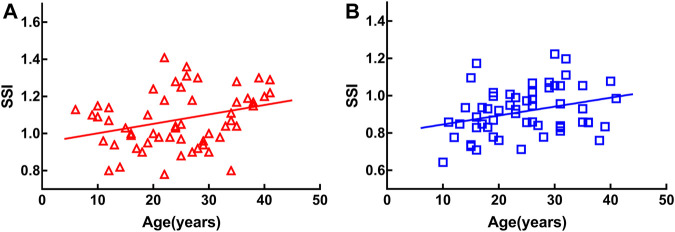
Stress-strain index (SSI) was positively correlated with age in both groups [moderately elongated eye: *r* = 0.326, *p* < 0.05 **(A)**, severely elongated eye: *r* = 0.298, *p* < 0.05 **(B)**].

SSI was positively correlated with age in both groups [MEE: *r* = 0.326, *p* < 0.05 (Figure 1A); SEE: *r* = 0.298, *p* < 0.05 (Figure 1B)].

**FIGURE 2:**
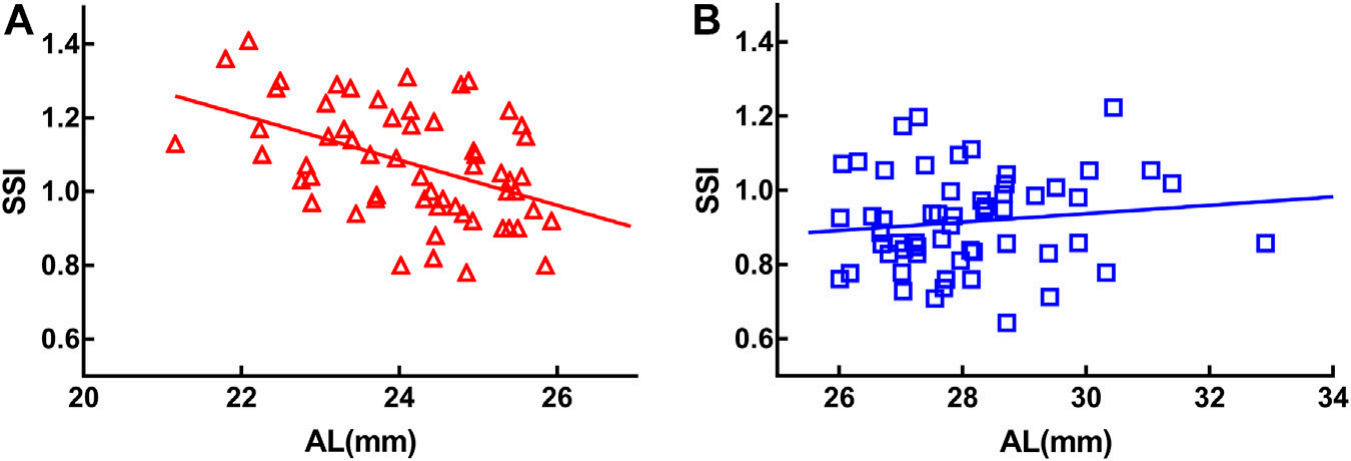
Stress-strain index (SSI) was negatively correlated with axial length (AL) in the moderately elongated eye group (*r* = −0.476, *p* < 0.001) **(A)**. There was no significant correlation between SSI and axial length in the severely elongated eye group (*p* > 0.05) **(B)**.

Age was used as a control variable in the following analyses. SSI was negatively correlated with AL in the MEE group (*r* = −0.476, *p* < 0.001) (Figure 2A) but not in the SEE group (*r* = 0.033, *p* = 0.809) (Figure 2B).

**FIGURE 3:**
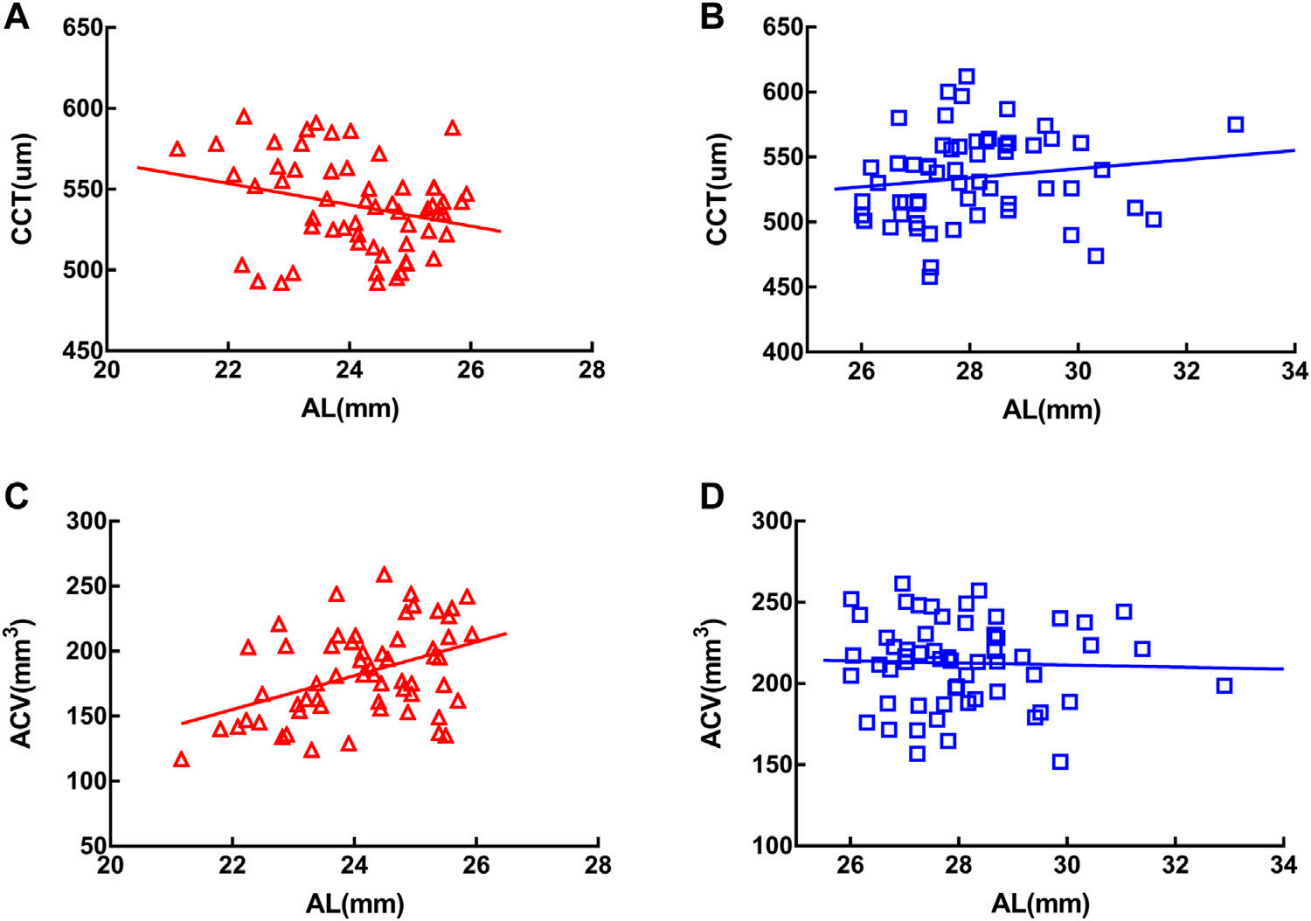
Central corneal thickness (CCT) was negatively correlated with axial length in the moderately elongated eye group (*r* = −0.289, *p* < 0.05) **(A)** and anterior chamber volume (ACV) was positively correlated with axial length (*r* = 0.444, *p* < 0.001) **(C)**. In the severely elongated eye group, neither CCT **(B)** nor ACV **(D)** was associated with axial length (*p* > 0.05).

In the MEE group, CCT was negatively correlated with AL (*r* = −0.289, *p* < 0.05) (Figure 3A) and ACV positively correlated with AL (*r* = 0.444, *p* < 0.001) (Figure 3C). In the SEE group, neither CCT (Figure 3B) nor ACV (Figure 3D) was correlated with AL (*p* > 0.05). Partial correlation coefficients adjusted for age between the clinical characteristics of the two groups were summarized in Table 4.

**TABLE 4:** Partial correlation coefficient adjusted for age between ocular parameters of the two groups.

Parameters	MEE		SEE	
	r	*p*	r	*p*
SSI vs. AGE	0.326^b^	<0.05^a^	0.298^b^	<0.05^a^
SSI vs. AL	−0.476^c^	<0.001^a^	-^c^	0.809
AL vs. CCT	−0.289^c^	<0.05^a^	-^c^	0.304
AL vs. ACV	0.444^c^	<0.001^a^	-^c^	0.900

a Statistically significant.

b Pearson correlation.

c Coefficient of partial correlation after adjusting for age, SSI, Stress-Strain index; AL, Axial length; CCT, Central corneal thickness; ACV, Anterior chamber volume; MEE, moderately elongated eye; SEE, severely elongated eye.

## Discussion

In this study, we investigated the relationship between the corneal stiffness parameter SSI and AL in a Chinese population. Overall we found that the eyes with longer AL had lower SSI. In the MEE group, AL was negatively correlated to the SSI. While in the SEE group, SSI was no longer correlated to AL.

Age is an important factor that affects corneal biomechanics (Blackburn et al., 2019). Previous studies have shown that CH, CRF, and deformation amplitude at highest concavity are related to age (Elsheikh et al., 2008a; Wang et al., 2017; Momeni-Moghaddam et al., 2019). In addition, the stiffness of the cornea increases with age (Elsheikh et al., 2010). Compared to CH (an indicator of the viscous properties of the cornea) and CRF (an indicator of the overall resistance or elastic properties of the cornea) (Nongpiur et al., 2015), SSI in this study reflects corneal stiffness (Eliasy et al., 2019). To verify the reliability of this conclusion, we analyzed the relationship between the SSI and age. Our results showed that although the SSI was more significantly reduced in the SEE group than in the MEE group, it was still positively correlated with age in both groups. This suggested that the cornea progressively stiffened with age, regardless of their AL. The fibrous stroma was the main layer that dominated the corneal biomechanical behavior (Boyce et al., 2008). With the presence of proteoglycan in the interfibrillar matrix, stromal microstructural components, including both collagen fibrils and fibrillar molecules, increase with age (Daxer et al., 1998; Elsheikh et al., 2008a).

Excessive axial elongation can lead to a higher myopic refractive error. This may cause excessive stretching of the retina, choroid, and sclera, which in turn result in a series of eye complications such as macular degeneration, retinal detachment, and posterior staphyloma (Ohno-Matsui and Jonas, 2019). In recent years, researchers have investigated the relationship between corneal biomechanics and axial elongation, and have arrived at various conclusions (Hon et al., 2017; Matalia et al., 2017; Long et al., 2019; Tubtimthong et al., 2020; Yu et al., 2020). Long et al. believed that spherical equivalent refraction was significantly positively correlated with the stiffness parameter at the first applanation (SP-A1) (Long et al., 2019). Bueno-Gimeno et al. found that lower CH and CRF were significantly associated with longer AL (Bueno-Gimeno et al., 2014). However, some studies have also shown that corneal biomechanics do not correlate with AL or refractive error (Inceoglu et al., 2018; Lim et al., 2008). Nevertheless, it is noteworthy that the biomechanical parameters provided by different instruments reflect different biomechanical properties of the cornea (Hon et al., 2017). In addition, it is believed that the range of selected myopic eyes might affect the correlation analysis between corneal biomechanics and AL (Del Buey et al., 2014). Therefore, in this study, we divided the subjects into groups according to the distribution of AL ranges to further investigate the relationship between SSI and AL.

In concordance with our previous findings, the SSI of the SEE group was significantly lower than that of the MEE group (Liu et al., 2020). In the MEE group, the SSI was significantly negatively correlated with AL. In contrast, no significant correlation was observed between the SSI and AL in the SEE group. This suggested that the effect of AL on corneal stiffness was not linear. We propose this could be due to the uneven growth of the eyeball. In the process of physiological myopia progression, axial elongation is relatively uniform and corresponds to the other components within the ocular structures. For instance, the crystalline lens thickness and corneal refractive power decrease in proportion to AL elongation (Meng et al., 2011). However, when the eye is elongated further, pathological changes start to appear (Backhouse and Phillips, 2010; Kang et al., 2018). When the eye progresses to a pathological state, the traction resulting from the AL elongation is mainly from the posterior pole with minimal or no collaboration with the other parts of the globe; hence, the changes in the anterior structures tend to be relatively stable and irrelevant to the excessive AL elongation (Larsen, 1971; Khokhar et al., 2018). Khokhar et al. suggested that the anterior and posterior segments of the eye with low myopia or ametropia were proportional to eye growth but disproportionate in high myopia (Khokhar et al., 2018). Research using 3D MRI of myopia morphology also found that the longitudinal and transverse diameter ratio of the eyeball was larger in high myopia than in non-high myopia (Wen et al., 2017). This suggested that the expansion of the eyeball was uneven in SEE as compared to MEE.

It has been revealed that scleral biomechanics may influence the axial elongation rate, with poor scleral biomechanics corresponding to a faster axial elongation rate (Haseltine et al., 2012; Hon et al., 2017). However, owing to the difficulty in measuring posterior scleral biomechanics *in vivo*, some researchers suggested measuring the anterior segment parameters instead of posterior scleral biomechanics to predict the development of myopia (Hayashi et al., 2010; Long et al., 2019; Wu et al., 2019). Bataille et al. investigated the relationship between AL and various anterior segment parameters and developed a model to predict AL; however, the author pointed out that a larger sample size was needed for validation (Bataille et al., 2021).

In the current study, we also found that CCT was negatively correlated with AL and ACV positively correlated with AL in MEE, whereas neither CCT nor ACV was correlated with AL in SEE. Similar findings have been reported in previous studies. Hashemi et al. found that anterior chamber depth plus crystalline lens thickness was associated with AL only in eyes with a shorter AL, but not in eyes with a longer AL (Hashemi et al., 2015). Cheng et al. found that when AL was less than 27 mm, the crystalline lens power was negatively correlated with AL, whereas in eyes with an AL greater than 27 mm, the crystalline lens power was no longer correlated with AL (Cheng et al., 2020). This also proved that in SEE, the correlation between the anterior segment and posterior pole of the eye are weakened. This may explain why the corneal SSI was no longer correlated with AL in SEE.

Previous studies suggested that corneal biomechanics could be used to evaluate the structural and functional glaucomatous damage, because the biomechanics of the sclera and lamina cribrosa could be represented by that of the cornea to some extent (Chansangpetch et al., 2017; Aoki et al., 2020; Miki et al., 2020). Hocaoglu et al. found that in patients with glaucoma whose ALs ranged from 21.10 to 25.51 mm, CH was negatively correlated with global retinal nerve fiber layer thickness. Aoki et al. showed that in glaucomatous eyes with 21.10–27.00 mm ALs, the optic nerve head (ONH) morphology was associated with the biomechanical properties measured using Corvis ST. Glaucomatous ONH superior-inferior asymmetries were associated with small deformations and slow recovery of the cornea (Aoki et al., 2020). Pillunat et al. believed that lower adjusted CH and CRF was negatively correlated with the severity of open-angle glaucoma in patients with ALs from 21.80 to 25.10 mm (Pillunat et al., 2016). Although these studies explored the relationship between corneal biomechanics and glaucoma, most of the selected participants had MEE. In this study, we found a lower correlation between corneal stiffness and posterior polar region morphology in SEE. Considering the correlation between corneal stiffness and glaucomatous changes in MEE, further studies are warranted on these parameters in patients with SEE.

## Limitations

A limitation of this research was that scleral biomechanics could not be directly measured to analyze the biomechanical correlation between the cornea and sclera in different AL groups. Whether the parameters of scleral mechanics can be reflected indirectly through the measurement of corneal biomechanical parameters has not been supported by this research, and warrants further investigation.

## Conclusion

To conclude, SSI, the corneal stiffness parameter was lower in the SEE group than in the MEE group. This study analyzed for the first time the correlation between SSI and AL in subjects with MEE and SEE. The SSI was negatively correlated with AL in the MEE group, but not in the SEE group. This may explain the controversial correlation between corneal stiffness and AL in previous studies and provide insights into the different eye growth pattern in lower myopia and higher myopia.

## Data Availability

The raw data supporting the conclusion of this article will be made available by the authors, without undue reservation.
